# Testing Control Strategies for Foot-and-Mouth Disease in New England Using the InterSpread Plus Model: Impacts of Regional Zoning, Early Detection, and Enhanced Biosecurity

**DOI:** 10.3390/v18040480

**Published:** 2026-04-21

**Authors:** Johnbosco U. Osuagwu, Julia M. Smith, Scott C. Merrill

**Affiliations:** 1 Department of Animal and Veterinary Sciences, College of Agriculture and Life Sciences, University of Vermont, Burlington, VT 05405, USA; 2 Department of Agriculture, Landscape, and Environment, College of Agriculture and Life Sciences, University of Vermont, Burlington, VT 05405, USA

**Keywords:** biosecurity, dairy industry, FMD, ISP+, regional zoning, simulation modeling

## Abstract

Foot-and-mouth disease (FMD) poses a significant threat to the United States dairy industry. This study evaluates the effectiveness of regional zoning, enhanced detection, and biosecurity in controlling FMD spread, focusing on the New England milkshed, using the InterSpread Plus (ISP+) model. We adapted a baseline ISP+ configuration incorporating United States dairy farm data, movement networks, cattle dealers, markets, and slaughterhouses, with milk processing plants as a model addition. Four hypotheses were tested to understand the impact of different biosecurity strategies: (H1) regional zoning limits the interregional spread of FMD post-detection; (H2) earlier detection in New England via increased passive surveillance reduces the overall outbreak impact; (H3) reduced indirect transmission through enhanced biosecurity measures improves FMD outbreak control; (H4) the combination of regional zoning and earlier detection provides synergistic reduction in FMD impact beyond either strategy alone. The four hypotheses were tested using three geographically distinct infection seed sets; 100 iterations of each scenario were simulated over 210 days and compared to the baseline. Key impact metrics included the daily number of infected premises, the outbreak duration, and the total number of infected premises across the outbreak scenarios. Results suggest shorter outbreak durations and reduced total infected premises under the hypothesized scenarios, compared to the baseline scenario. Kruskal–Wallis H tests confirmed significant differences across the baseline, regional zoning, early detection, enhanced biosecurity, and the combination of heightened passive surveillance with regional zoning scenarios in terms of total infected premises. Post hoc Dunn’s tests indicated that enhanced biosecurity outperformed other control strategies tested. These findings demonstrate that layered interventions may substantially curtail both the national amplification and local spread of FMD, and thus protect the consumer milk supply and reduce cascading economic shocks from an outbreak.

## 1. Introduction

Foot-and-mouth disease (FMD) is a highly contagious viral infection affecting cloven-hoofed animals, including cattle, pigs, sheep, goats, and deer, and posing a severe threat to global livestock industries [1,2,3,4]. Caused by the FMD virus (FMDV), a member of the *Picornaviridae* family and *Aphthovirus* genus, FMD historically has manifested through seven immunologically distinct serotypes (O, A, C, SAT 1, SAT 2, SAT 3, and Asia 1), each with multiple subtypes, complicating control efforts due to limited cross-immunity [1,3,4,5]. Serotype O is the most prevalent globally, responsible for major epidemics such as the pan-Asian outbreak starting in 1990, while serotype C has become rare, with no isolations since 2004 [1,6]. The virus’s stability in various environments—surviving up to three months in manure or six months in cold soil, and only inactivating under extreme pH or temperatures—enhances its persistence and transmission potential [2,7].

Transmission occurs via multiple routes, including direct contact with infected animals’ secretions and excretions (e.g., saliva, milk, urine, feces, semen), indirect contact through contaminated fomites (vehicles, equipment, feed), and aerosols, which can travel up to 60 km over land or 300 km over water under favorable conditions [2,7,8]. Pigs amplify aerosol spread, while cattle are highly susceptible via inhalation [2,7]. Mechanical vectors, such as humans carrying the virus on clothing or skin, and contaminated animal products further facilitate dissemination [2,8]. Carrier states in ruminants—lasting up to 3.5 years in cattle and five years in African buffalo—pose ongoing risks, though transmission from carriers to naive animals is debated and primarily documented in African SAT serotypes [2]. Incubation periods range from hours to 14 days, during which subclinical shedding may allow the virus to spread before clinical signs are detected, complicating early identification and control [9].

Clinically, FMD presents with fever and vesicular lesions on the mouth, feet, and teats, leading to salivation, lameness, reduced feed intake, abortions, and decreased milk yield in chronically affected animals [10,11]. Mortality is low in adults but higher in neonates due to myocarditis [10,11]. In dairy cattle, persistent low milk production and secondary infections exacerbate losses [12]. Symptomatically similar diagnoses include vesicular stomatitis and footrot, necessitating laboratory confirmation [9,10,11].

Globally, FMD is endemic in parts of Africa, Asia, the Middle East, and South America, causing cyclical outbreaks that disrupt production and trade [13,14]. Economic impacts are profound: direct losses from reduced meat and milk output, mortality, and control costs, coupled with indirect effects like trade embargoes, can total billions of dollars [13,14]. The 2001 UK outbreak cost over £8 billion, highlighting vulnerabilities in FMD-free regions [13]. In endemic areas, FMD constrains agricultural development and food security, with annual global losses estimated at several billion USD [13].

In the United States, FMD was eradicated in 1929. The country has since maintained FMD-free status without vaccination, which is crucial for livestock exports [15]. However, the United States remains vulnerable due to global trade, smuggling, and livestock movements [15,16,17]. A hypothetical outbreak could cost $15–$228 billion, depending on scope, from production halts, culling, and trade bans [18,19].

The United States dairy industry, valued at approximately $40 billion annually, is a critical component of the agricultural economy, producing over 223 billion pounds of milk and exporting approximately 15% of its dairy production [20]. Dairy farms operate within complex supply chains involving frequent animal movements, milk transport, feed, manure management, and interactions with processing facilities [20]. The interconnected nature of United States dairy operations necessitates robust control strategies to mitigate the risk of an FMD outbreak.

Control strategies for FMD include stamping-out (culling infected herds), vaccination, movement restrictions, and zoning [1,21,22,23,24]. Stamping-out is standard in FMD-free countries but is costly and ethically challenging [1]. Vaccination, while effective for matched serotypes, delays immunity and may allow carrier states, complicating trade resumption [2]. Regional zoning—dividing areas into compartments based on disease status—enables targeted controls and continuity of business in free zones, as per the World Organization for Animal Health guidelines [23]. This approach accounts for livestock structures, movements, and risks, facilitating trade from unaffected areas [2,21,23,25]. Though regional zoning strategies have been successfully implemented in some European Union countries—where supranational coordination allows uniform movement controls across national borders—their application in the United States dairy industry remains underexplored. In the United States, dairy operations fall under state-level authority—with no interstate border controls managed by federal agencies and no standardized interstate checkpoints between states—creating unique jurisdictional fragmentation that complicates coordinated regional standstill rules.

Epidemiological modeling plays a pivotal role in evaluating control efficacy, predicting spread, and informing policy [18,19,26,27,28,29]. Simulation models like the Animal Disease Spread Model and InterSpread Plus (ISP+) enable scenario testing [27,28]. ISP+, a spatially explicit, stochastic model, simulates between-farm spread using farm-level data, contact networks, and controls [28]. Developed from InterSpread [29] and used in the 2001 United Kingdom (UK) FMD response, the ISP+ incorporates stochastic processes for infection, detection, and interventions, making it versatile for diseases like FMD, avian influenza, and classical swine fever [28]. It requires inputs on farm locations, animal populations, movement types, and parameters for transmission probabilities, allowing evaluation of zoning, surveillance, and biosecurity.

The dairy industry in New England exemplifies regional vulnerabilities. Comprising six states, Connecticut, Maine, Massachusetts, New Hampshire, Rhode Island, and Vermont (that is, CT, ME, MA, NH, RI, and VT, respectively), it produces high milk volumes (about 24,000 lbs/cow annually) and tallies a high number of on-farm and off-farm processing plants in the region, but faces economic distress, fragmented regulations, and dense interconnections [30,31]. Farms are family-owned, averaging about 100 cows on 400 acres, with cooperatives and processors handling interstate milk transport [30,31]. The risks associated with the movements of animals (high risk for FMD) and animal products (medium–high risk for FMD), as well as heightened interconnectivity due to density, amplify spread potential [30,32]. Economic pressures—low profitability, off-farm work, development threats—risk farm closures post-outbreak, while regulatory silos across states hinder unified responses [30,32]. Wildlife reservoirs (e.g., deer, bison) and agritourism add layers of risk and further complicate response [30].

Despite FMD’s absence, the characteristics of New England’s dairy sector—interstate dependencies, small-scale operations, and an emphasis on local foods—heighten vulnerability [30,32]. Literature gaps include region-specific modeling of zoning, detection, and biosecurity in dense milksheds of the New England region. Moreover, traditional national strategies may falter due to intensified local indirect contacts (e.g., via milk haulers and processors) and limited cross-regional movement data, potentially leading to underestimated local amplification and delayed containment.

This study addresses these gaps using ISP+ to evaluate FMD control in New England’s dairy industry. The objectives are: (1) to adapt a baseline ISP+ model incorporating United States dairy data, movements (milk plants, dealers, processors), and FMD parameters; (2) to test four hypotheses: New England regional zoning reduces the overall FMD impact in the United States by limiting cross-zone spread; earlier detection in the New England region via enhanced passive surveillance reduces the overall outbreak impact; reduced indirect transmission in the New England region through enhanced biosecurity improves FMD control; and the combination of regional zoning and earlier detection provides synergistic reduction in FMD impact beyond either strategy alone; (3) to analyze outcomes in terms of infected premises and duration, with sensitivity testing.

## 2. Methodology

This study employed a simulation-based approach to evaluate control strategies for FMD in the New England dairy industry using the ISP+ model [28], which integrates epidemiological parameters, spatial data, and control interventions to simulate between-premises transmission dynamics. The model operates on a state-transition basis, where dairy premises progress through states such as susceptible, infected, clinical, detected, and depopulated, influenced by probabilistic events. Transmission pathways include direct contacts (live animal movements), indirect contacts (e.g., via vehicles or personnel), local spread, and airborne dissemination.

ISP+ has been widely applied in veterinary epidemiology for diseases like FMD, avian influenza, and classical swine fever, enabling policy evaluation through scenario testing [28]. Its stochastic nature accounts for variability, with multiple iterations providing distributions of outcomes. For this study, ISP+ version 6.2.1.55 was used, configured for the United States dairy sector with a focus on New England.

### 2.1. Data Sources and Farm File Configuration

**Figure 1:**
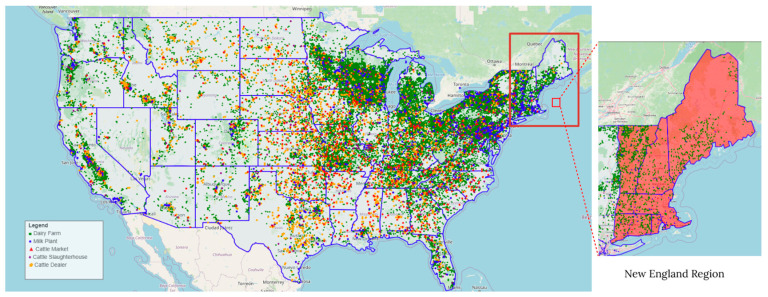
Livestock premises in the United States (with blue state boundaries) modeled with ISP+ (New England region highlighted), comprising dairy farms, milk processing plants, cattle markets, cattle slaughterhouses, and cattle dealers.

The model utilized a comprehensive farm file representing United States livestock premises generated primarily from the Farm Location and Animal Population Simulator [33], using data sourced from the USDA National Agricultural Statistics Service (NASS) 2022 database, state agricultural reports, and regional milkshed mappings [34,35,36]. As an innovation, the locations of milk processing plants were integrated into the base model. This was done by obtaining the plants’ longitudes and latitudes from the USDA Federal Milk Marketing Orders [36], converting them to the USA Contiguous Albers Equal Area Conic coordinate reference system (see Supplementary Figure S1), and modifying the Albers reference system to ensure positive values for the northing and easting coordinates readable by the ISP+ model. This modified farm file configuration comprised 39,670 premises, consisting of 35,975 dairy farms, 3031 cattle dealers, 1033 cattle markets, 663 cattle slaughterhouses, and 696 milk processing plants, as shown in Figure 1.

Each seeding set infected three farms on day 1, with detections on days 9–11 to reflect hypothetical FMD incursions. Each iteration only used one of the three seed sets. Seed Set 1 featured mixed locations: two farms near New England (New York) and one distant farm (in Tennessee); Seed Set 2 placed all three introductions in New York, proximal to the New England region; Seed Set 3 used three distant southwestern sites (one farm in California and two farms in Arizona).

User-defined states included the following: depopulated, tracing states (direct/indirect contact), heightened local spread, and surveillance phases. An additional state for post-depopulation spread was also included, such that there was a residual risk for FMD transmission from infected premises with delayed depopulation following detection. Furthermore, a regional zone state was defined for the New England region. This state enforced movement restrictions across the zone boundaries once triggered, representing an innovation in the ISP+ model that allowed explicit geographic zoning at the New England regional scale beyond standard national control area zoning.

### 2.2. Baseline Model Configuration

Table 1 summarizes the movement source, movement distance, transmission probability, and movement destination parameters used to configure the ISP+ baseline model to simulate FMD transmission across various United States dairy operations. In the baseline scenario, time period triggers defined seasonal variations (winter, spring, summer, autumn, repeating annually) and post-detection phases (comprising movement standstill 1–3 days post-detection and a post-standstill reduction in the probability of movement thereafter).

Movement types encompassed direct and indirect contacts, tailored to dairy operations: dairy farms, cattle markets, cattle slaughterhouses, cattle dealers, and milk movements to various milk processing plants. Frequencies followed Poisson distributions of the number of movements per day per premises category (e.g., 0.0321 for large dairy, 0.0043 for medium dairy, 0.0004 for small). Some milk movement parameters were based on expert opinion from discussions with researchers, veterinary epidemiologists, and infectious disease modelers.

The probabilities of movement to a destination premises varied by the source premises (e.g., 0.5249 for large dairy movements destined for a cattle dealer). The distances of such movements used binned probabilities (e.g., 0–10 km: low probability for long-haul).

Table 2 summarizes the infectivity, local spread, airborne spread, and surveillance zone parameters used to configure the ISP+ baseline model to simulate FMD transmission across various United States dairy operations. Transmission probabilities employed BetaPert distributions (e.g., 0.02–0.08 for market infections) or tables for time-dependent infectivity. Indirect movements included medium-/low-risk categories, differentiated by premises categories and their FMD detection status.

Local spread was modeled at heightened states for 25% of large and medium dairy farms; 50% of small dairy farms, cattle markets, and cattle slaughterhouses; and 10% of milk processing plants. Airborne spread was minimized and was based on seasonal directional weightings and very low cattle transmission probabilities (that is, 0.0134 within 1 km and 0 beyond a 1 km distance).

Control measures included movement restrictions (e.g., 50% standstill for livestock, 25% for milk post-detection post-standstill). Tracing was required for all direct contacts and indirect medium-risk contacts with an FMD-detected premises, with BetaPert delays (e.g., 0–7–14 days). Surveillance on most premises was passive post-detection, with detection probability tables (e.g., 1.0–0.0 over days), and active for premises in the detected or buffer zones (e.g., visit frequency and duration with BetaPert 112). Depopulation and disposal followed detection; post-depopulation states were included.

The outputs for each iteration captured infected, clinical, detected, depopulated, traced, and zoned farms, as well as movements and outbreak duration.

### 2.3. Scenario Development for Hypotheses

Four hypotheses were tested against the baseline model configuration described in Table 1 and Table 2, adjusting certain effective FMD contact parameters to simulate enhanced FMD controls in the New England region. The model originally consisted of five geographic regions: Pacific Coast, Mountain West, Great Lakes, Southeast, and Northeast, determined by observed intrastate and interstate movement tendencies occurring within and between specific regions. To extract the premises in the New England region, individual coordinate files for each state in the Northeast were integrated into the model, and the six states within the New England region (that is CT, ME, MA, NH, RI, and VT) were sub-grouped as a regional zone. A time-point trigger was then integrated into the New England regional zone of the model such that once the first FMD case was detected anywhere in the United States, no live animals or raw milk could move into the New England regional zone—essentially a regional standstill that halted cross-boundary traffic while allowing intra-zone movements to continue under standard national control area rules.

**Hypothesis 1** **(H1).** *New England regional zoning reduces the overall FMD impact in the United States by limiting FMD spread arising from interregional movements.*

**Hypothesis 2** **(H2).** *Earlier detection of FMD in the New England region via enhanced passive surveillance reduces the overall outbreak impact.*

To simulate earlier detection via enhanced passive surveillance in the New England region, the detection probability of the dairy premises within this region was increased by 20% (that is, a multiplier of 1.2 compared to the baseline) to reflect intensified monitoring in the New England milkshed following an outbreak of FMD in the United States.

**Hypothesis 3** **(H3).** *Reduced indirect transmission in the New England region through enhanced biosecurity improves FMD control.*

On the assumption that enhanced biosecurity lowers FMD transmission risks, a ‘Set State’ parameter for enhanced biosecurity, representing a 50% reduction in the baseline FMD transmission probability for low- and moderate-risk indirect contacts, was integrated into the model, applied to 90% of all the premises within the New England region, and triggered following the first detection of FMD in the United States.

**Hypothesis 4** **(H4).** *The combination of regional zoning and earlier detection (via heightened passive surveillance) in New England provides a synergistic reduction in FMD impact beyond either strategy alone by coupling movement restrictions with earlier detection triggers.*

To simulate the combined heightened passive surveillance with zoning scenarios, the integrated time-point trigger, which specifically limited the movement of live animals and raw milk from dairy premises into the New England region post-first-detection of FMD in the United States, was combined with the 20% increased detection probability of the dairy premises within the region.

### 2.4. Simulation Parameters and Execution

The FMD transmission parameters, like the incubation period, clinical duration, infectivity peak, Poisson distribution for movements, and BetaPert distributions for FMD detection delays and probabilities, were incorporated in initialization files.

The InterSpread Plus model was executed for 100 independent stochastic iterations for each of the five scenarios (that is, the baseline scenario and the four hypothesized scenarios) within each of the three seed sets, for a total of 1500 simulations (3 seed sets × 5 scenarios × 100 iterations), each run for 210 days. Outcomes were explicitly measured at the national (whole United States) and regional (New England-only) scales. Seed Set 1, with mixed proximity, seeded infection at two farms close to New England and one away from the New England region. Seed Set 2 seeded infection at three farms proximal to the New England region, thus plausibly enabling rapid dissemination into the New England region. Seed Set 3 seeded infection in three distant farms unlikely to infect New England within the simulation timeframe. The five scenarios were: (i) baseline (with no additional New England-specific controls), (ii) New England regional zoning, (iii) early detection via heightened passive surveillance, (iv) enhanced biosecurity (50% reduction in indirect transmission probabilities post-detection), and (v) the New England region combining zoning with heightened passive surveillance.

### 2.5. Outcome Measures

The primary outcomes measured at the national level and the New England regional level were the daily number of infected premises, the outbreak duration (days to last detection), and the total number of infected premises across the outbreak scenarios.

### 2.6. Statistical and Sensitivity Analysis

To compare the differences in FMD outbreak outcomes between the baseline model and the hypothesized model scenarios, the Kruskal–Wallis H test for non-parametric distributions across scenarios was applied [45]. Post hoc Dunn’s tests to compute the pairwise differences across scenarios were then calculated [46].

Statistical analysis was completed in Python (version 3.10.11) with ‘statsmodels’, with alpha (significance) set at *p* < 0.05 [47] (see Supplementary Python Code).

## 3. Results and Interpretation of Results

### 3.1. Total Infected Premises Outcomes Across Seed Sets and Scenarios

**Figure 2:**
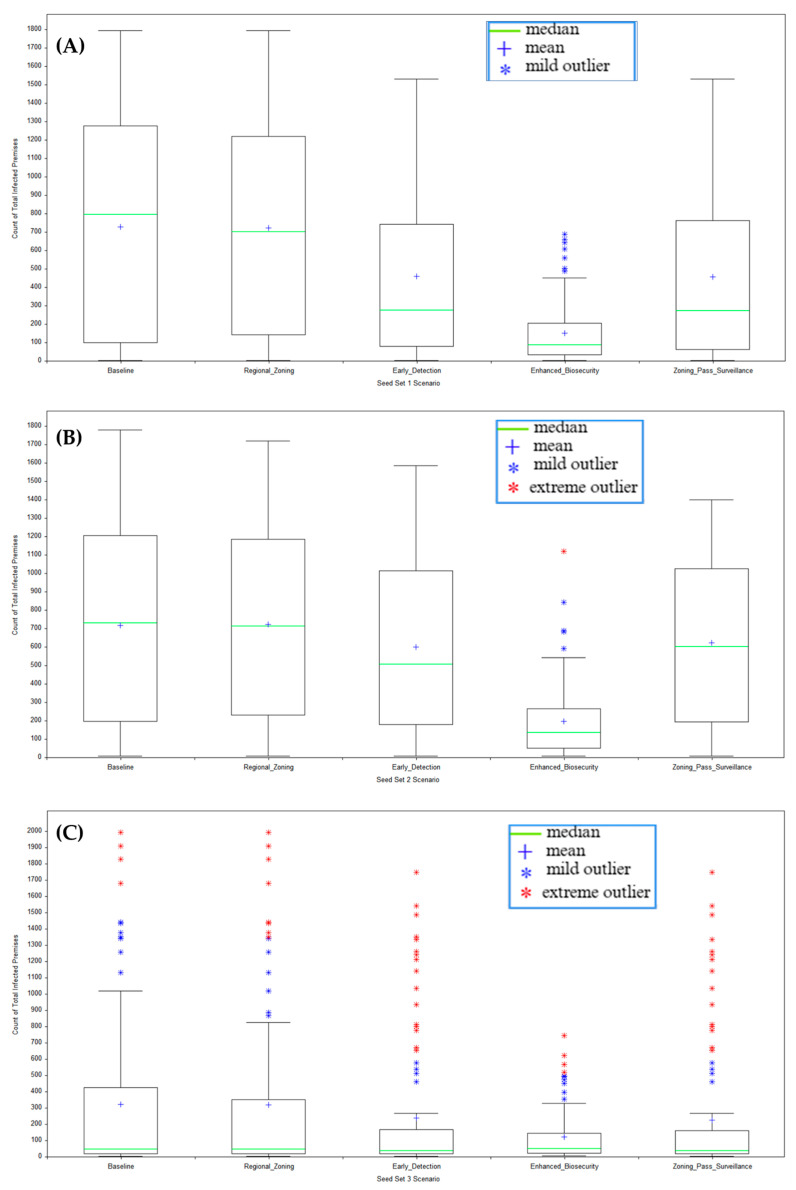
Total infected premises across Seed Sets 1 (**A**), 2 (**B**), and 3 (**C**) scenarios (the whole United States).

Across Seed Sets 1 and 2, as shown in Figure 2, the enhanced biosecurity scenario produced the lowest national median total infected premises and, critically, the narrowest interquartile ranges, reflecting the model’s direct multiplicative attenuation of indirect low-risk and medium-risk movement probabilities combined with immediate activation of heightened local spread mitigation states. In Seed Set 1 (mixed proximity), with enhanced biosecurity, the national median fell to 88 premises (25th–75th percentile range 33–206, IQR approximately 173), compared with the baseline median of 799 (100–1262, IQR ~1162), regional zoning median of 702 (148–1211, IQR ~1794), early detection median of 278 (83–742, IQR ~1531) and combined heightened passive surveillance with zoning median of 273 (66–755, IQR ~1531). Seed Set 2 (all introductions proximal to the New England region), with enhanced biosecurity, showed a biosecurity median of 139 (53–265, IQR ~212) versus baseline 732 (207–1203, IQR ~996), regional zoning median of 716 (231–1180, IQR ~949), early detection median of 509 (182–1005, IQR ~823) and combined heightened passive surveillance with zoning median of 602 (196–1024, IQR ~828). In Seed Set 3 (distant southwestern sites), where absolute infection numbers remained low, enhanced biosecurity with a national median of 47 (25th–75th percentile range 35–60) did not appear to outperform any of the other scenarios in terms of median infected total infected premises. However, biosecurity maintained the tightest distribution at IQR ~25, suggesting that it had the lowest potential across all scenarios to incur larger outbreak sizes.

**Figure 3:**
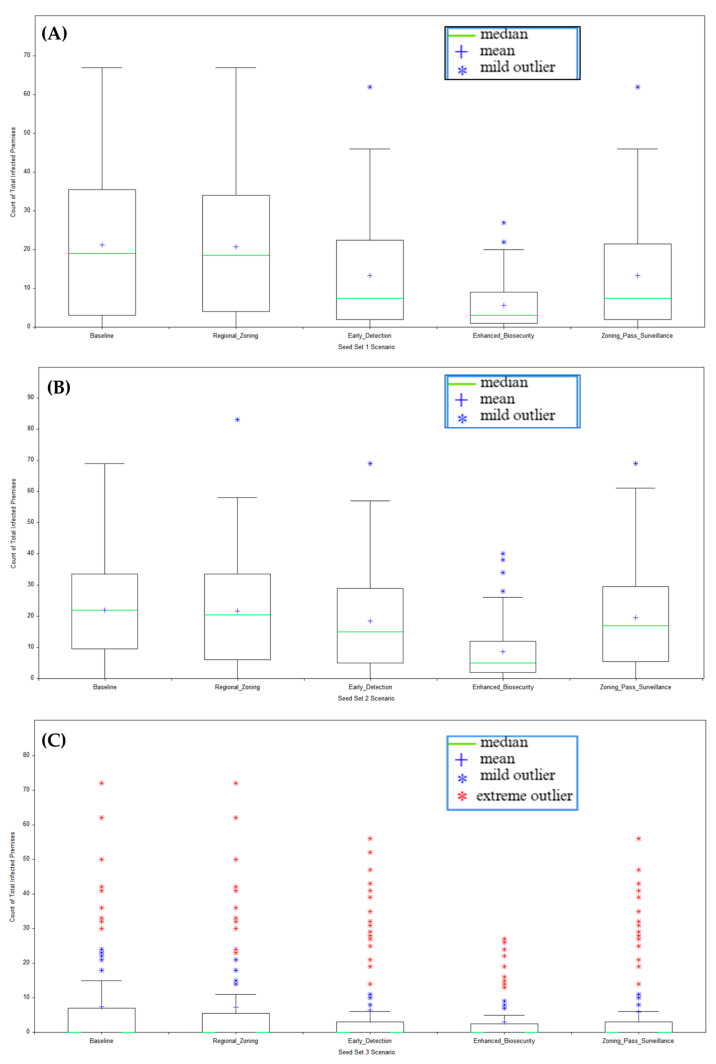
Total infected premises across Seed Sets 1 (**A**), 2 (**B**), and 3 (**C**) scenarios (New England region only).

New England-only metrics shown in Figure 3 reveal little to no infections in the New England region in Seed Set 3 (distant southwestern sites), with a median of zero infected premises across all scenarios. In the highest-risk local-introduction case (Seed Set 2), biosecurity capped New England spread at a median of seven premises (25th–75th percentile range 4–15, IQR ~11), versus baseline 23 (12–35, IQR ~23), regional zoning 22 (10–34, IQR ~24), early detection 16 (6–31, IQR ~25), and combined heightened passive surveillance with zoning median of 21 (8–32, IQR ~24) premises.

### 3.2. Epidemic Curve Dynamics Across Scenarios

**Figure 4:**
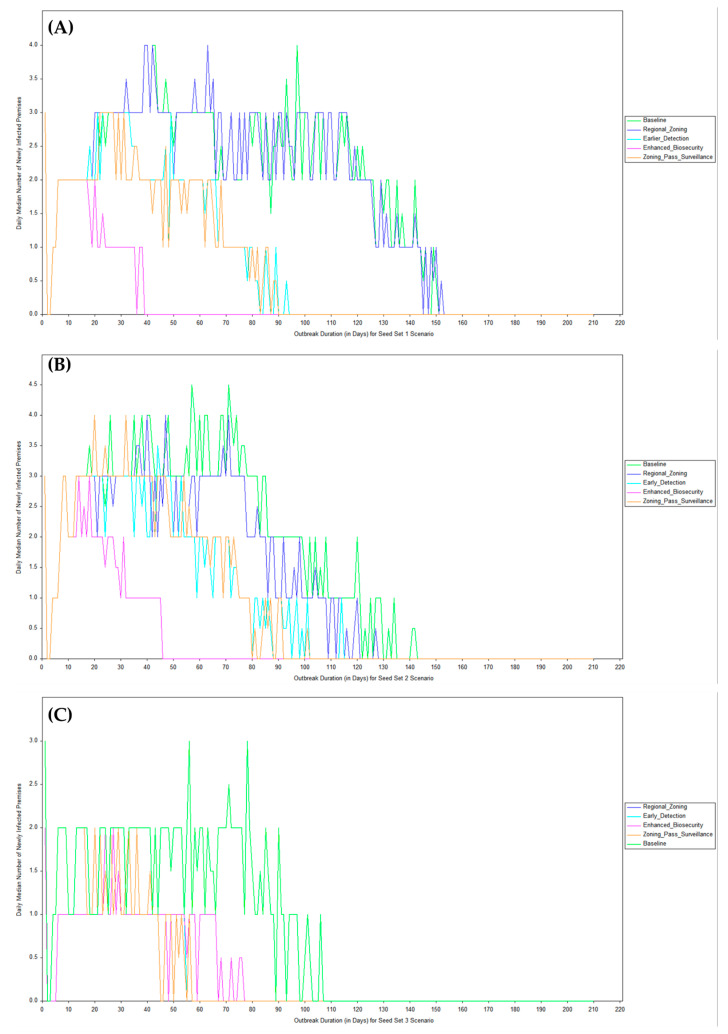
Epidemic curves for Seed Sets 1 (**A**), 2 (**B**), and 3 (**C**), across the modeled baseline, regional zoning, early detection, and increased passive surveillance combined with zoning scenarios.

Daily median newly infected premises curves in Figure 4 exposed spatial–temporal interactions. In Seed Set 1 (mixed introductions), baseline and zoning curves exhibited prolonged plateaus. However, compared to the regional zoning scenario, where the high level of median daily infections began declining around day 70, the high level of median daily infections in the baseline scenario persisted till about day 100 (that is, an extra 30 days). The newly infected premises under the enhanced biosecurity scenario declined around day 18 and flattened sharply after day 40, rarely exceeding 1–2 new premises daily. The early detection and the heightened passive surveillance combined with zoning scenarios also produced an outbreak decline after day 35 and flattened after days 90 and 95, respectively.

Seed Set 2 (introductions proximal to New England) generated the highest baseline peaks (daily median > five premises around day 60. Baseline and zoning curves appeared similar. However, the regional zoning scenario had an earlier peak, shorter plateau, and shorter duration than the baseline scenario. The newly infected premises under the enhanced biosecurity scenario declined around day 20 and flattened sharply after day 39, rarely exceeding 1–2 new premises daily. The early detection and the heightened passive surveillance combined with zoning scenarios produced similar outbreak curves, declining after day 35 and flattening after day 104. Seed Set 3 (distant southwestern introductions) showed lower overall curves across all scenarios, with relative flattening under biosecurity, early detection, and heightened passive surveillance combined with zoning scenarios remaining similar.

As shown in Figure 4, the interventions often shifted the epidemic curves leftward and downward, reducing both duration and cumulative area under the curve.

### 3.3. Statistical Comparisons

Kruskal–Wallis H tests for Seed Sets 1 and 2 confirmed significant differences (*p* < 0.001) in total infected premises across the baseline, regional zoning, earlier detection, enhanced biosecurity, and combined heightened passive surveillance with zoning scenarios. The Dunn’s post hoc comparisons (Bonferroni-adjusted) isolated biosecurity’s superiority over every comparator. Even in Seed Set 3, where national differences in total infected premises were non-significant (*p* > 0.05) across scenarios, biosecurity’s New England-only subset retained *p* < 0.001. This suggested that enhanced biosecurity delivered strong localized protection without necessarily altering the nationwide outbreak size when introductions occurred far from the New England regional zone.

### 3.4. Sensitivity Analysis

**Figure 5:**
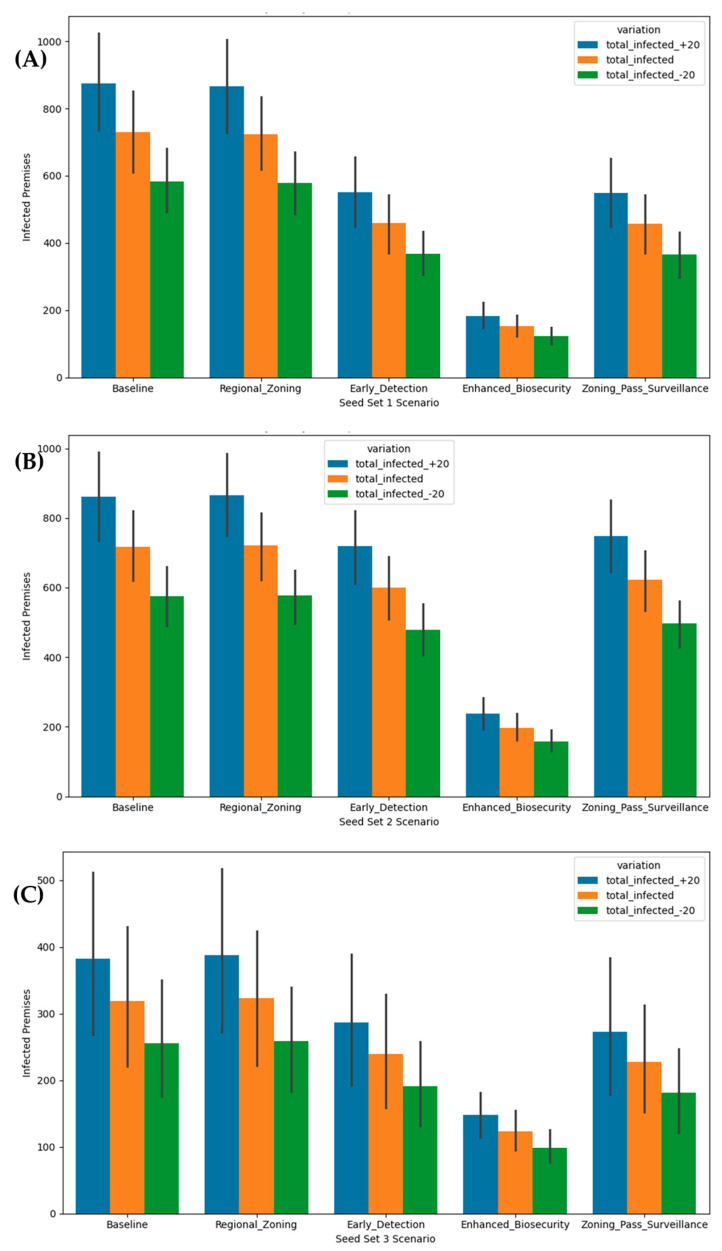
Sensitivity analysis across Seed Sets 1 (**A**), 2 (**B**), and 3 (**C**) scenarios, to evaluate the impact of heightened and reduced infectivity using ±20% transmission rates.

To evaluate robustness, transmission rates were varied by ±20%. Under +20% (heightened infectivity), the medians of total infected premises scaled proportionally, with the baseline scenario resulting in 959, 879, and 57 total infected premises for Seed Sets 1, 2, and 3 respectively; the regional zoning scenario resulting in 842, 859, and 57 total infected premises for Seed Sets 1, 2, and 3 respectively; the early detection scenario resulting in 334, 611, and 48 total infected premises for Seed Sets 1, 2, and 3 respectively; the enhanced biosecurity scenario resulting in 105, 167, and 48 total infected premises for Seed Sets 1, 2, and 3 respectively; and the combined heightened passive surveillance with zoning scenario resulting in 328, 722, and 48 total infected premises for Seed Sets 1, 2, and 3 respectively. For −20% (reduced infectivity), the baseline scenario resulted in 639, 586, and 38 total infected premises for Seed Sets 1, 2, and 3 respectively; the regional zoning scenario resulted in 562, 573, and 38 total infected premises for Seed Sets 1, 2, and 3 respectively; the early detection scenario resulted in 223, 407, and 32 total infected premises for Seed Sets 1, 2, and 3 respectively; the enhanced biosecurity scenario resulted in 70, 111, and 32 total infected premises for Seed Sets 1, 2, and 3 respectively; and the combined heightened passive surveillance with zoning scenario resulted in 219, 482, and 32 total infected premises for Seed Sets 1, 2, and 3 respectively. Kruskal–Wallis tests remained highly significant (H = 69.46, *p* < 0.001) for both infectivity variations, indicating consistent relative benefits. Figure 5 illustrates these medians of the total infected premises, showing enhanced biosecurity’s superior resilience across parameter shifts.

## 4. Discussion

The simulation outcomes highlight the differential efficacy of regional zoning, early detection, and enhanced biosecurity in controlling FMD in the United States dairy industry, with enhanced biosecurity emerging as particularly potent in curbing outbreak scale. These findings align with epidemiological tenets emphasizing interruption of transmission pathways in high-density regions [2]. By integrating stochastic elements, the study captures real-world variability, offering insights for policy in FMD-free nations vulnerable to reintroduction [15].

Regional zoning’s modest reduction in total infected premises validates the value of layered movement restrictions in limiting FMD amplification via interregional movements, particularly in the New England region with dense dairy networks [30,48]. By sub-grouping states and triggering post-detection restrictions, regional zoning contained spread, and in some scenarios, reduced median outbreak durations by 30% (that is, from 100 days to 70 days). This aligns with the Australian Animal Disease Spread Model that explores the possibility of zoning as an optional FMD control strategy to support trade [49]. In the United States context, where exports hinge on FMD-free status, zoning minimizes trade embargoes, potentially averting direct and indirect annual losses [13]. Regional zoning alone contributed a relatively small incremental benefit in most seed sets, underscoring that its greater impact may occur when paired with other control strategies such as earlier detection via increased passive surveillance.

Early detection’s reduction in the total infected premises underscores surveillance’s role in truncating FMD transmission [9,25]. The 20% probability boost in the early detection scenario simulated intensified monitoring, aligning with findings that earlier detection significantly influences FMD outbreak control and spread [39]. In our results of the early detection scenario compared to the baseline scenario, the peak day and peak count of infected premises fell. Economically, this peak infection drop translates to averted losses in production and trade disruptions [13].

Enhanced biosecurity’s marked impact stems from halving indirect transmission risks post-detection, targeting fomites, vehicles, and personnel common in livestock operations [2]. This mirrors the enhanced biosecurity framework, which emphasizes operational indicators, such as restricting entry to essential personnel, controlling wildlife access, and sanitizing equipment, as critical for FMD exclusion in high-risk dairy environments [50]. The narrower IQR and lower minima in infected premises suggest that enhanced biosecurity could abort outbreaks early in favorable iterations, consistent with experimental studies showing reduced fomite persistence under enhanced protocols [7]. However, outbreak outcome variability following biosecurity may indicate its sensitivity to compliance and timing; its delayed implementation could allow initial amplification, as seen in some scenarios with higher maxima.

The markedly smaller absolute infection scale observed in Seed Set 3—despite the southern United States containing large absolute numbers of dairy operations—could be a direct consequence of ISP+’s distance-dependent transmission kernel interacting with real-world dairy density gradients. The seeded southern states typically span vast landscapes with more dairy premises but spread over enormous land area, placing far fewer premises within the effective 0–1000 m and 1–3 km transmission radii. In contrast, New England’s dairy premises are concentrated in a much tighter geographic footprint, amplifying local spread when introductions occur nearby. This density effect is not an artifact of the model but a verified epidemiological reality: USDA NASS 2024–2025 state-level inventories confirm New England and mid-Atlantic states maintain higher farms-per-square-kilometer values than southwestern states, precisely the spatial configuration that drives higher baseline medians in Seed Sets 1 and 2.

Synergies among strategies exist. Zoning, when combined with increased passive surveillance, yielded better outbreak outcomes in most scenarios compared to the baseline, regional zoning only, or early detection only. This supports European Union and South American directives blending zoning, surveillance, and biosecurity [25,51]. However, when introductions are proximal to the New England region (as seen in Seed Set 2), such that infections could potentially spread into the zone before detection, regionalization combined with increased passive surveillance may be effectively similar to early detection alone. A key strength of our ISP+ adaptation is the explicit inclusion of milk processing plants as networked premises, enabling realistic simulation of dairy-specific transmission via shared tankers and equipment—pathways often underrepresented (and their outbreak impact consequently underestimated) in generic livestock models. This creates opportunities for future efforts, such as modeling targeted interventions at plants (e.g., disinfection protocols) or integrating real-time GPS data for dynamic hauler movements, potentially refining risk assessments in fragmented supply chains.

Limitations encompass model assumptions: movement data may underestimate long-distance events, and airborne spread was minimized despite its potential role [2,8]. Expert opinion on some milk movement parameters was necessary because publicly available quantitative data on some indirect contacts (e.g., indirect movements from milk processing plants) and New England-specific biosecurity practices remain limited. Stochasticity captured variability, but unmodeled factors, like carrier states or compliance, could inflate risks [2]. To satisfy the modeling need to effectively compare the baseline scenario to the hypothesized scenarios while adjusting for the location of risk introduction, the initial infections were seeded on the same three farms per location set and detected on the same days, and local spread was heightened on the same proportion of dairy premises. Therefore, the results are not generalizable to other outbreaks that could begin at different locations, be detected at different times, or have different proportions of heightened local spreaders. The parameter estimates for infectivity, local spread, and airborne spread are based on FMDV serotype O, which presents a high risk for incursion into the United States [1,39]. Though sensitivity analysis confirmed the model’s robustness to varying infectivity, which could be likened to varying serotype infectivity, future work could incorporate strain-specific parameters.

These results advocate preemptive zoning plans, surveillance investments, and biosecurity protocols to fortify the United States dairy industry against FMD, minimizing economic and production losses. However, modeling the cost associated with undertaking these advocated strategies could be integrated into future work to get a clearer picture of their cost–benefit analyses. While the present study focused on epidemiological outcomes, a formal cost-effectiveness analysis of the tested strategies—incorporating direct culling costs, trade losses, and intervention expenses—would further strengthen policy recommendations.

## 5. Conclusions

Foot-and-mouth disease remains one of the most economically devastating livestock diseases worldwide, capable of triggering trade embargoes, widespread culling, and supply-chain disruptions that could cost the United States dairy industry significant economic and production losses. This study used the spatially explicit InterSpread Plus model to evaluate targeted regional zoning, earlier detection, and enhanced biosecurity control strategies in the New England region, using three geographically distinct seed sets. Across all sets, enhanced biosecurity consistently delivered the largest reductions in infected premises (80–89% nationally) with the narrowest interquartile ranges, while regional zoning and heightened passive surveillance together provided a strong synergistic FMD containment.

These findings demonstrate that layered interventions—built on the ISP+ radial kernel, dairy-specific movement networks, and regional zoning rules—may substantially curtail both national amplification and local spread. Regionalization could emerge as the practical path forward for New England: geographic boundary-based restrictions could enable disease-free subpopulations to maintain trade continuity under the World Organization for Animal Health standards while preserving the interstate milk flows that supply regional processing needs. By limiting interregional movements post-detection and applying enhanced biosecurity, the modeled strategies could support some degree of continuity of business during FMD outbreaks.

Investments in regional zoning plans, surveillance infrastructure, and enhanced on-farm biosecurity protocols are therefore warranted now. Future research should quantify the cost–benefit trade-offs of these combined strategies, test variable compliance levels, and incorporate real-time dairy movement data to further strengthen national preparedness against this economically devastating disease.

The significance of these results extends far beyond the New England dairy sector. Family-scale operations, dense processing plants, agritourism activities, and interstate interconnections make the milkshed uniquely vulnerable; yet the same factors make early, regionally tailored controls exceptionally effective. Successful implementation of regional zoning and biosecurity could protect the consumer milk supply and reduce cascading economic shocks. In short, regional preparedness is not merely a technical exercise—it is a safeguard for an entire industry and the communities that depend on it. By acting on the evidence presented here, policymakers, producers, and stakeholders can translate simulation outcomes into resilient dairy industry defenses that preserve both animal health and economic vitality.

## Figures and Tables

**Table 1:** Parameters for movement types in FMD transmission among United States dairy operations.

Movement Source Name	Movement Distance (km Bins and Probabilities)	Probability of Transmission	Movement Destination Name (with Probabilities)	Data Source
large_dairy_farm (dairy_l)	0: 0, 500: 0.867, 1000: 0.067, 2000: 0.049, 4500: 0.017	0.0378 min, 0.9999 max; tabled over days	* dairy_l: 0.4035, dairy_s: 0.118, cattle_dealer: 0.5249, dairy_m: 0.0598	[37]
medium_dairy_farm (dairy_m)	0: 0, 500: 0.867, 1000: 0.067, 2000: 0.049, 4500: 0.017	0.1125 min, 0.8473 max; tabled over days	* dairy_l: 0.2584, dairy_s: 0.0413, cattle_dealer: 0.5022, dairy_m: 0.1981	[37]
small_dairy_farm (dairy_s)	0: 0, 500: 0.867, 1000: 0.067, 2000: 0.049, 4500: 0.017	0.0055 min, 0.9999 max; tabled over days	* dairy_l: 0.4035, dairy_s: 0.0118, cattle_dealer: 0.5249, dairy_m: 0.0598	[37]
dairy_movement_to_infect_market	0: 0, 500: 0.676, 1000: 0.15, 2000: 0.13, 4500: 0.044	BetaPert (0.02–0.04–0.08)	* cattle_market: 1.0	[37]
cattle_markets	0: 0, 500: 0.726, 1000: 0.137, 2000: 0.109, 4500: 0.0264	0.0087 min, 0.9999 max; tabled over days	* dairy_l: 0.5580, dairy_s: 0.0555, cattle_dealer: 0.1508, dairy_m: 0.2357	[37]
large_dairy_cattle_through_market	0: 0, 500: 0.726, 1000: 0.137, 2000: 0.109, 4500: 0.0264	BetaPert (0.02–0.04–0.08)	* cattle_market: 1.0	[37]
cattle_movement_that_infects_cattle_slaughterhouses	0: 0, 125: 0.7886, 250: 0.1064, 375: 0.0255, 500: 0.0618, 625: 0.0135, 750: 0.0035, 875: 0.0003, 1000: 0.0003, 2011+: 0.0001	BetaPert (0.02–0.04–0.08)	small_cattle_slaughterhouses: 0.8, large_cattle_slaughterhouses: 0.2	[38]
cattle_dealer	0: 0, 100: 0.83577, 200: 0.1129, 300: 0.02311, 400: 0.01195, 500: 0.01259, 600: 0.00299, 700: 0.00027, 800: 0.00037, 900+: 0.00005	0.4500 min, 0.999 max; tabled over days	dairy_l: 0.7706, dairy_s: 0.0401, dairy_m: 0.1893	[38]
indirect_movement_from_cattle_market_to_premise	0: 0, 500: 0.726, 1000: 0.139, 2000: 0.109, 4500: 0.026	BetaPert (0.02–0.04–0.08)	dairy_l: 0.4624, dairy_s: 0.0461, cattle_dealer: 0.2972, dairy_m: 0.1943	[38]
indirect_movement_from_cattle_slaughterhouses	0: 0, 125: 0.7886, 250: 0.1064, 375: 0.0255, 500: 0.0618, 625: 0.0135, 750: 0.0035, 875: 0.0003, 1000: 0.0003, 2011+: 0.0001	BetaPert (0.000125–0.00025–0.0005)	dairy_l: 0.2814, dairy_s: 0.0902, cattle_dealer: 0.5123, dairy_m: 0.1161	[38]
indirect_medium_risk_movement_North_East	0: 0, 60: 0.9572, 80: 0.0231, 100: 0.0034, 200: 0.0093, 2000+: 0.007	0 min, 0.5 max; tabled over days	** varying probability distributions depending on source	[37]
indirect_medium_risk_movement_other_regions	0: 0, 60: 0.9572, 80: 0.0231, 100: 0.0034, 200: 0.0093, 2000+: 0.007	0 min, 0.5 max; tabled over days	** varying probability distributions depending on source	[37]
indirect_low_risk_movement_North_East	0: 0, 60: 0.9839, 80: 0.0115, 200: 0.0023, 2000+: 0.0023	0 min, 0.1 max; tabled over days	** varying probability distributions depending on source	[37]
indirect_low_risk_movement_other_regions	0: 0, 60: 0.9839, 80: 0.0115, 200: 0.0023, 2000+: 0.0023	0 min, 0.1 max; tabled over days	** varying probability distributions depending on source	[37]
indirect_detected_dairy_premises	0: 0, 40: 0.94, 80: 0.05, 160+: 0.01	0 min, 0.01 max; tabled over days	varying probability distributions depending on source	[38]
milk_movement_from_large_dairy	0: 0, 100: 0.5, 321: 0.35, 804: 0.1, 900+: 0.05	0 min, 0.1 max; tabled over days	** dairy_l: 0.05, LMP: 0.60, SMP: 0.25, PHP: 0.10	[37]
milk_movement_from_medium_dairy	0: 0, 100: 0.3, 321: 0.5, 804: 0.15, 900+: 0.05	0 min, 0.1 max; tabled over days	** dairy_s: 0.10, LMP: 0.20, SMP: 0.40, PHP: 0.20 dairy_m: 0.10	[37]
milk_movement_from_small_dairy	0: 0, 100: 0.3, 321: 0.5, 804: 0.15, 900+: 0.05	0 min, 0.1 max; tabled over days	** dairy_s: 0.10, LMP: 0.2, SMP: 0.35, PHP: 0.15 dairy_m: 0.2	[37]
milk_movement_from_small_milkplants (SMP)	0: 0, 100: 0.6, 321: 0.3, 804: 0.05, 900+: 0.05	0 min, 0.1 max; tabled over days	dairy_s: 0.10, LMP: 0.50, PHP: 0.20 dairy_m: 0.20	Expert opinion
milk_movement_from_large_milkplants (LMP)	0: 0, 100: 0.6, 321: 0.3, 804: 0.05, 900+: 0.05	0 min, 0.1 max; tabled over days	dairy_s: 0.10, SMP: 0.55, PHP: 0.20 dairy_m: 0.15	Expert opinion
milk_movement_from_producer_handler_plants (PHP)	0: 0, 100: 0.6, 321: 0.3, 804: 0.05, 900+: 0.05	0 min, 0.1 max; tabled over days	dairy_s: 0.05, dairy_l: 0.05, LMP: 0.35, SMP: 0.50 dairy_m: 0.05	Expert opinion

* represents the percentage of destinations for permanently removed cows, by herd size and region. ** represents the percentage of operations on which visitors had animal contact, by herd size and region.

**Table 2:** Parameters for infectivity, local spread, airborne spread, and surveillance zones in FMD transmission among United States dairy operations.

Parameter	Value(s)	Reference(s)
maximum time of infectiousness	(in days)	[39]
dairy_l, dairy_s, cattle_dealer	BetaPert (30–34–42)	[40]
cattle market, cattle slaughterhouse	BetaPert (7–10–14)	[41]
milkplant	BetaPert (1–4–28)	Expert opinion
infection to clinical signs onset (day: probability)	0: 0, 1: 0.1209, 2: 0.2940, 3: 0.5046, 4: 0.6968, 5: 0.8372, 6: 0.9225, 7: 0.9670, 8: 0.9873, 9: 1	[8]
local spread probability of transmission following onset of clinical signs	(day:km bins:probability)	[42]
when not detected and not heightened	0:1:0, 0:2:0, 0:3:0, 0:4:0, 1:1:0.007, 1:2:0.002, 1:3:0, 1:4:0, 2:1:0.012, 2:2:0.003, 2:3:0.001, 2:4:0, 3:1:0.012, 3:2:0.004, 3:3:0.001, 3:4:0	[42]
when not detected and heightened	0:1:0, 0:2:0, 0:3:0, 0:4:0, 1:1:0.007, 1:2:0.002, 1:3:0, 1:4:0, 2:1:0.012, 2:2:0.003, 2:3:0.001, 2:4:0, 3:1:0.015, 3:2:0.0044, 3:3:0.001, 3:4:0	[42]
when detected and not depopulated and not heightened	0:1:0, 0:2:0, 0:3:0, 0:4:0, 1:1:0.00175, 1:2:0.0005, 1:3:0, 1:4:0, 2:1:0.003, 2:2:0.00075, 2:3:0.00025, 2:4:0, 3:1:0.003, 3:2:0.001, 3:3:0.00025, 3:4:0	[42]
when detected and not depopulated and heightened	0:1:0, 0:2:0, 0:3:0, 0:4:0, 1:1:0.00175, 1:2:0.0005, 1:3:0, 1:4:0, 2:1:0.003, 2:2:0.00075, 2:3:0.00025, 2:4:0, 3:1:0.0375, 3:2:0.0011, 3:3:0.00025, 3:4:0	[42]
when depopulated and no post-depopulation spread and not heightened	0:1:0, 0:2:0, 0:3:0, 0:4:0, 1:1:0.000875, 1:2:0.00025, 1:3:0, 1:4:0, 2:1:0.0015, 2:2:0.000375, 2:3:0.000125, 2:4:0, 3:1:0.0015, 3:2:0.0005, 3:3:0.000125, 3:4:0	[42]
when depopulated and no post-depopulation spread and heightened	0:1:0, 0:2:0, 0:3:0, 0:4:0, 1:1:0.000875, 1:2:0.00025, 1:3:0, 1:4:0, 2:1:0.0015, 2:2:0.000375, 2:3:0.000125, 2:4:0, 3:1:0.001875, 3:2:0.00055, 3:3:0.000125, 3:4:0	[42]
when not detected and no post-depopulation spread	0–7:20:0.000005, 8:20:0, 0–8:20+:0	[42]
airborne spread (km bins: probability)	1: 0.00118, 1+: 0	[43]
surveillance zones	(in km radii from infected premises)	[44]
infected premises detection zone	0.005	[28]
control area	10	[44]
surveillance zone	20	[44]

## Data Availability

The data supporting the case study are available on request from the corresponding author due to privacy reasons.

## Supplementary Materials

The following supporting information can be downloaded at: https://www.mdpi.com/article/10.3390/v18040480/s1, Figure S1: Showing the USA Contiguous Albers Equal Area Conic Map of Premises Locations.

## Abbreviations

The following abbreviations are used in this manuscript:

FMD	Foot-and-mouth disease
ISP+	InterSpread Plus
FMDV	Foot-and-mouth disease virus
UK	United Kingdom
NASS	National Agricultural Statistics Service
IQR	Interquartile range
