# Supplementary material for: Testing Control Strategies for Foot-and-Mouth Disease in New England Using the InterSpread Plus Model: Impacts of Regional Zoning, Early Detection, and Enhanced Biosecurity
Source: Viruses. 2026 Apr 21;18(4):480. doi: 10.3390/v18040480 (PMC13119767; doi:10.3390/v18040480)
Supplement: Supplementary file 1 [file viruses-18-00480-s001.zip › viruses-4263130-supplementary.pdf]

1. **PYTHON (version 3.10.11) CODE:** Comparing the outbreak outcomes across the baseline, regional zoning, early detection, enhanced biosecurity, and the combination of heightened passive surveillance with regional zoning scenarios in the InterSpread Plus model, and then statistically testing the outcome differences using the Kruskal-Wallis H tests and Post-Hoc Dunn's tests.

```
import pandas as pd
import numpy as np
from scipy import stats
import matplotlib.pyplot as plt
import os
import seaborn as sns
import itertools
from statsmodels.stats.multitest import multipletests # For Bonferroni correction if needed

# Try importing scikit-posthocs, fall back to manual if not available
try:
    import scikit_posthocs as sp
    USE_DUNN = True
except ImportError:
    USE_DUNN = False
    print("scikit-posthocs not available. Using manual pairwise Mann-Whitney U tests with
Bonferroni correction for post-hoc.")

# Function to parse a log file into a DataFrame
def parse_log_file(file_path, scenario_name):
    data = []
    try:
        with open(file_path, 'r') as file:
            for line in file:
                parts = line.split()
                if len(parts) >= 6:  # Minimum expected fields
                    iteration = int(parts[0])
                    day = int(parts[1])
                    farm_id = parts[2]
                    movement_type = parts[3]
                    coord_y = float(parts[4])
                    coord_x = float(parts[5])
                    data.append({
                        'iteration': iteration,
                        'day': day,
                        'farm_id': farm_id,
                        'movement_type': movement_type,
                        'coord_y': coord_y,
                        'coord_x': coord_x,
                        'scenario': scenario_name
                    })
    except FileNotFoundError:
        print(f"Error: File {file_path} not found.")
        return pd.DataFrame()
    return pd.DataFrame(data)

# Define file paths
file_paths = {
    'Baseline': 'Baseline_1.txt',
    'Early_Detection': 'Earlier_Detection_1.txt',
    'Regional_Zoning': 'Regional_Zoning_1.txt',
    'Enhanced_Biosecurity': 'Enhanced_Biosecurity_1.txt',
    'Zoning_Pass_Surveillance': 'Zoning_Earlier_Detection_1.txt'
}

# Parse files for each scenario
dataframes = {scenario: parse_log_file(path, scenario) for scenario, path in
file_paths.items()}

# Combine
df_all = pd.concat([df for df in dataframes.values() if not df.empty], ignore_index=True)
```

```
# Define New England bounding box (assuming coord_x = lon, coord_y = lat)
min_lon = -73.73
max_lon = -66.95
min_lat = 40.98
max_lat = 47.46

# Filter for New England
df_all['is_ne'] = ((df_all['coord_x'] >= min_lon) & (df_all['coord_x'] <= max_lon) &
                   (df_all['coord_y'] >= min_lat) & (df_all['coord_y'] <= max_lat))
df_ne = df_all[df_all['is_ne']].copy()

print("Extracted New England Infections:")
print(df_ne)

# Optionally save to CSV
df_ne.to_csv('new_england_infections_1D2.csv', index=False)
print("New England infections saved to 'new_england_infections_1D2.csv'")

# Now proceed with calculations for the entire United States (all), and perhaps for New
England (NE) separately

# For all
daily_infected = df_all.groupby(['scenario', 'iteration',
'day'])['farm_id'].nunique().reset_index(name='daily_infected')

total_infected = daily_infected.groupby(['scenario',
'iteration'])['daily_infected'].sum().reset_index(name='total_infected')

total_infected['affected_cattle'] = total_infected['total_infected'] * 50

outbreak_duration = daily_infected.groupby(['scenario',
'iteration'])['day'].max().reset_index(name='duration')

total_infected['depopulated'] = total_infected['total_infected'] * 0.8

total_infected['traced'] = total_infected['total_infected'] * 2

metrics = total_infected.merge(outbreak_duration, on=['scenario', 'iteration'])

# Summary for all
summary = metrics.groupby('scenario').agg({
    'total_infected': ['min', lambda x: np.percentile(x, 25), 'median', lambda x:
np.percentile(x, 75), 'max', lambda x: np.percentile(x, 75) - np.percentile(x, 25)],
    'affected_cattle': ['min', lambda x: np.percentile(x, 25), 'median', lambda x:
np.percentile(x, 75), 'max', lambda x: np.percentile(x, 75) - np.percentile(x, 25)],
    'duration': ['min', lambda x: np.percentile(x, 25), 'median', lambda x: np.percentile(x,
75), 'max', lambda x: np.percentile(x, 75) - np.percentile(x, 25)],
    'depopulated': ['min', lambda x: np.percentile(x, 25), 'median', lambda x:
np.percentile(x, 75), 'max', lambda x: np.percentile(x, 75) - np.percentile(x, 25)],
    'traced': ['min', lambda x: np.percentile(x, 25), 'median', lambda x: np.percentile(x,
75), 'max', lambda x: np.percentile(x, 75) - np.percentile(x, 25)]
}).reset_index()

summary.columns = [
    'scenario',
    'infected_min', 'infected_25', 'infected_median', 'infected_75', 'infected_max',
'infected_iqr',
    'cattle_min', 'cattle_25', 'cattle_median', 'cattle_75', 'cattle_max', 'cattle_iqr',
    'duration_min', 'duration_25', 'duration_median', 'duration_75', 'duration_max',
'duration_iqr',
    'depop_min', 'depop_25', 'depop_median', 'depop_75', 'depop_max', 'depop_iqr',
    'traced_min', 'traced_25', 'traced_median', 'traced_75', 'traced_max', 'traced_iqr'
]

pd.set_option('display.max_columns', None)
pd.set_option('display.width', None)
pd.set_option('display.float_format', '{:.2f}'.format)

print("Summary Metrics (All):")
print(summary)

# Summary For NE
if not df_ne.empty:
```

```
    daily_infected_ne = df_ne.groupby(['scenario', 'iteration',
'day'])['farm_id'].nunique().reset_index(name='daily_infected')

    total_infected_ne = daily_infected_ne.groupby(['scenario',
'iteration'])['daily_infected'].sum().reset_index(name='total_infected')

    total_infected_ne['affected_cattle'] = total_infected_ne['total_infected'] * 50

    outbreak_duration_ne = daily_infected_ne.groupby(['scenario',
'iteration'])['day'].max().reset_index(name='duration')

    total_infected_ne['depopulated'] = total_infected_ne['total_infected'] * 0.8

    total_infected_ne['traced'] = total_infected_ne['total_infected'] * 2

    metrics_ne = total_infected_ne.merge(outbreak_duration_ne, on=['scenario', 'iteration'])

    summary_ne = metrics_ne.groupby('scenario').agg({
        'total_infected': ['min', lambda x: np.percentile(x, 25), 'median', lambda x:
np.percentile(x, 75), 'max', lambda x: np.percentile(x, 75) - np.percentile(x, 25)],
        'affected_cattle': ['min', lambda x: np.percentile(x, 25), 'median', lambda x:
np.percentile(x, 75), 'max', lambda x: np.percentile(x, 75) - np.percentile(x, 25)],
        'duration': ['min', lambda x: np.percentile(x, 25), 'median', lambda x:
np.percentile(x, 75), 'max', lambda x: np.percentile(x, 75) - np.percentile(x, 25)],
        'depopulated': ['min', lambda x: np.percentile(x, 25), 'median', lambda x:
np.percentile(x, 75), 'max', lambda x: np.percentile(x, 75) - np.percentile(x, 25)],
        'traced': ['min', lambda x: np.percentile(x, 25), 'median', lambda x:
np.percentile(x, 75), 'max', lambda x: np.percentile(x, 75) - np.percentile(x, 25)]
    }).reset_index()

    summary_ne.columns = [
        'scenario',
        'infected_min', 'infected_25', 'infected_median', 'infected_75', 'infected_max',
'infected_iqr',
        'cattle_min', 'cattle_25', 'cattle_median', 'cattle_75', 'cattle_max', 'cattle_iqr',
        'duration_min', 'duration_25', 'duration_median', 'duration_75', 'duration_max',
'duration_iqr',
        'depop_min', 'depop_25', 'depop_median', 'depop_75', 'depop_max', 'depop_iqr',
        'traced_min', 'traced_25', 'traced_median', 'traced_75', 'traced_max', 'traced_iqr'
    ]

    print("Summary Metrics (New England Only):")
    print(summary_ne)

# Epidemic Peak for all
peak_infected = daily_infected.groupby(['scenario',
'iteration'])['daily_infected'].max().reset_index(name='peak_infected')
metrics = metrics.merge(peak_infected, on=['scenario', 'iteration'])

peak_summary = metrics.groupby('scenario')['peak_infected'].agg(['min', lambda x:
np.percentile(x, 25), 'median', lambda x: np.percentile(x, 75), 'max']).reset_index()
peak_summary.columns = ['scenario', 'peak_min', 'peak_25', 'peak_median', 'peak_75',
'peak_max']

print("\nEpidemic Peak Summary (All):")
print(peak_summary)

# Epidemic Peak For NE
if not df_ne.empty:
    peak_infected_ne = daily_infected_ne.groupby(['scenario',
'iteration'])['daily_infected'].max().reset_index(name='peak_infected')
    metrics_ne = metrics_ne.merge(peak_infected_ne, on=['scenario', 'iteration'])

    peak_summary_ne = metrics_ne.groupby('scenario')['peak_infected'].agg(['min', lambda x:
np.percentile(x, 25), 'median', lambda x: np.percentile(x, 75), 'max']).reset_index()
    peak_summary_ne.columns = ['scenario', 'peak_min', 'peak_25', 'peak_median', 'peak_75',
'peak_max']

    print("\nEpidemic Peak Summary (New England Only):")
    print(peak_summary_ne)

# Kruskal-Wallis and Post-hoc for all
scenarios = ['Baseline', 'Early_Detection', 'Regional_Zoning', 'Enhanced_Biosecurity',
'Zoning_Pass_Surveillance']
```

```
metrics_list = ['total_infected', 'affected_cattle', 'duration', 'depopulated', 'traced',
'peak_infected']

kw_results = {}
posthoc_results = {}

for metric in metrics_list:
    data_groups = [metrics[metrics['scenario'] == s][metric].values for s in scenarios if not
metrics[metrics['scenario'] == s].empty]
    if len(data_groups) == len(scenarios):
        h_stat, p_val = stats.kruskal(*data_groups)
        kw_results[metric] = {'H': round(h_stat, 3), 'p': f"{p_val:.2e}"}

        # Post-hoc
        if USE_DUNN:
            df_post = metrics[['scenario', metric]].copy()
            dunn = sp.posthoc_dunn(df_post, val_col=metric, group_col='scenario',
p_adjust='bonferroni')
            posthoc_results[metric] = dunn
        else:
            from itertools import combinations
            pairs = list(combinations(scenarios, 2))
            p_values = []
            for s1, s2 in pairs:
                _, p = stats.mannwhitneyu(metrics[metrics['scenario']==s1][metric],
                                          metrics[metrics['scenario']==s2][metric],
                                          alternative='two-sided')
                p_values.append(p)
            reject, p_corr, _, _ = multipletests(p_values, alpha=0.05, method='bonferroni')
            posthoc_df = pd.DataFrame({'pair': [f"{p[0]}-vs-{p[1]}" for p in pairs],
'p_uncorr': p_values, 'p_corr': p_corr, 'significant': reject})
            posthoc_results[metric] = posthoc_df
    else:
        kw_results[metric] = {'H': np.nan, 'p': np.nan}

print("\nKruskal-Wallis Results (All):")
print(pd.DataFrame(kw_results).T)

print("\nPost-hoc Results (All):")
for metric, res in posthoc_results.items():
    print(f"\n--- {metric} ---")
    print(res)

# Repeat for NE if not empty
if not df_ne.empty:
    kw_results_ne = {}
    posthoc_results_ne = {}

    for metric in metrics_list:
        data_groups = [metrics_ne[metrics_ne['scenario'] == s][metric].values for s in
scenarios if not metrics_ne[metrics_ne['scenario'] == s].empty]
        if len(data_groups) == len(scenarios):
            h_stat, p_val = stats.kruskal(*data_groups)
            kw_results_ne[metric] = {'H': round(h_stat, 3), 'p': f"{p_val:.2e}"}

            # Post-hoc
            if USE_DUNN:
                df_post = metrics_ne[['scenario', metric]].copy()
                dunn = sp.posthoc_dunn(df_post, val_col=metric, group_col='scenario',
p_adjust='bonferroni')
                posthoc_results_ne[metric] = dunn
            else:
                from itertools import combinations
                pairs = list(combinations(scenarios, 2))
                p_values = []
                for s1, s2 in pairs:
                    _, p = stats.mannwhitneyu(metrics_ne[metrics_ne['scenario']==s1][metric],
                                              metrics_ne[metrics_ne['scenario']==s2][metric],
                                              alternative='two-sided')
                    p_values.append(p)
                reject, p_corr, _, _ = multipletests(p_values, alpha=0.05,
method='bonferroni')
                posthoc_df = pd.DataFrame({'pair': [f"{p[0]}-vs-{p[1]}" for p in pairs],
'p_uncorr': p_values, 'p_corr': p_corr, 'significant': reject})
                posthoc_results_ne[metric] = posthoc_df
```

```
        else:
            kw_results_ne[metric] = {'H': np.nan, 'p': np.nan}

    print("\nKruskal-Wallis Results (New England Only):")
    print(pd.DataFrame(kw_results_ne).T)

    print("\nPost-hoc Results (New England Only):")
    for metric, res in posthoc_results_ne.items():
        print(f"\n--- {metric} ---")
        print(res)

# Epidemic Curves Plot for all
daily_median = daily_infected.groupby(['scenario',
'day'])['daily_infected'].median().reset_index()

plt.figure(figsize=(12, 7))
for scenario in scenarios:
    scenario_data = daily_median[daily_median['scenario'] == scenario]
    if not scenario_data.empty:
        plt.plot(scenario_data['day'], scenario_data['daily_infected'], label=scenario,
                 linewidth=2, marker='o' if scenario == 'Baseline' else None)

plt.xlabel('Outbreak Duration (Days)')
plt.ylabel('Daily Median Number of Infected Premises')
plt.title('Epidemic Curves Across Scenarios (All)')
plt.legend()
plt.grid(True, which='both', linestyle='--', alpha=0.7)
plt.xticks(np.arange(0, daily_median['day'].max() + 5, 5))
plt.yticks(np.arange(0, daily_median['daily_infected'].max() + 5, 5))
plt.tight_layout()
plt.savefig('ISPno_NE1D_Hypotheses_recoded_output_duration_70_daysIQR_all.png')
plt.close()

# Epidemic Curves Plot for NE
if not df_ne.empty:
    daily_median_ne = daily_infected_ne.groupby(['scenario',
'day'])['daily_infected'].median().reset_index()

    plt.figure(figsize=(12, 7))
    for scenario in scenarios:
        scenario_data = daily_median_ne[daily_median_ne['scenario'] == scenario]
        if not scenario_data.empty:
            plt.plot(scenario_data['day'], scenario_data['daily_infected'], label=scenario,
                     linewidth=2, marker='o' if scenario == 'Baseline' else None)

    plt.xlabel('Outbreak Duration (Days)')
    plt.ylabel('Daily Median Number of Newly Infected Premises')
    plt.title('Epidemic Curves Across Scenarios (New England Only)')
    plt.legend()
    plt.grid(True, which='both', linestyle='--', alpha=0.7)
    plt.xticks(np.arange(0, daily_median_ne['day'].max() + 5, 5))
    plt.yticks(np.arange(0, daily_median_ne['daily_infected'].max() + 5, 5))
    plt.tight_layout()
    plt.savefig('ISP_NE1D_Hypotheses_recoded_output_duration_70_daysIQR_ne.png')
    plt.close()

# Box Plot for all
plt.figure(figsize=(10, 6))
metrics.boxplot(column='total_infected', by='scenario', grid=False)
plt.title('Total Infected Premises Across Scenarios (100 Iterations) (All)', fontsize=11)
plt.xlabel('Scenario')
plt.ylabel('Count of Total Infected Premises')
plt.suptitle('')
max_y = metrics['total_infected'].max()
plt.yticks(np.arange(0, max_y + 100, 100))
plt.grid(True, axis='y', linestyle='--', alpha=0.7)
plt.tight_layout()
plt.savefig('ISP_no_NE1D_Hypotheses_output_recodedIQR_all.png')
plt.close()

# Box Plot for NE
if not df_ne.empty:
    plt.figure(figsize=(10, 6))
    metrics_ne.boxplot(column='total_infected', by='scenario', grid=True)
```

```
    plt.title('Total Infected Premises Across Scenarios (100 Iterations) (New England Only)',
fontsize=11)
    plt.xlabel('Scenario', fontsize=11)
    plt.ylabel('Count of Total Infected Premises')
    plt.suptitle('')
    max_y_ne = metrics_ne['total_infected'].max()
    plt.yticks(np.arange(0, max_y_ne + 10, 5))
    plt.grid(True, axis='y', linestyle='--', alpha=0.7)
    plt.tight_layout()
    plt.savefig('ISP_NE1D_Hypotheses_output_recodedIQR_ne.png')
    plt.close()

# Sensitivity Analysis for all
sensitivity = metrics.copy()
sensitivity['total_infected_+20'] = sensitivity['total_infected'] * 1.2
sensitivity['total_infected_-20'] = sensitivity['total_infected'] * 0.8
# Compute medians for sensitivity variations
sens_summary = sensitivity.groupby('scenario')[['total_infected_+20', 'total_infected_-
20']].median().reset_index()
print(sens_summary)
# === FORCE DESIRED ORDER FOR SENSITIVITY BARPLOT ===
desired_order = ['Baseline', 'Regional_Zoning', 'Early_Detection', 'Enhanced_Biosecurity',
'Zoning_Pass_Surveillance']
kw_sens = {}
for var in ['total_infected_+20', 'total_infected_-20']:
    data_groups = [sensitivity[sensitivity['scenario'] == s][var] for s in scenarios if not
sensitivity[sensitivity['scenario'] == s].empty]
    if len(data_groups) == len(scenarios):
        h_stat, p_val = stats.kruskal(*data_groups)
        kw_sens[var] = {'H': round(h_stat, 3), 'p': f"{p_val:.2e}"}

print("\nSensitivity Kruskal-Wallis Results (All):")
print(kw_sens)

sensitivity_melted = pd.melt(sensitivity, id_vars=['scenario'],
value_vars=['total_infected_+20', 'total_infected', 'total_infected_-20'],
                             var_name='variation', value_name='infected_count')

sensitivity_melted['scenario'] = pd.Categorical(sensitivity_melted['scenario'],
                                                categories=desired_order,
                                                ordered=True)
plt.figure(figsize=(10, 6))
sns.barplot(x='scenario', y='infected_count', hue='variation', data=sensitivity_melted)
plt.title('Sensitivity Analysis: Impact of ±20% Transmission Rate (All)')
plt.xlabel('Seed Set 1 Scenario')
plt.ylabel('Infected Premises')
plt.tight_layout()
plt.savefig('sensitivity_no_NE1D_analysisIQR_all.png')
plt.close()

# Sensitivity Analysis for NE
if not df_ne.empty:
    sensitivity_ne = metrics_ne.copy()
    sensitivity_ne['total_infected_+20'] = sensitivity_ne['total_infected'] * 1.2
    sensitivity_ne['total_infected_-20'] = sensitivity_ne['total_infected'] * 0.8

    kw_sens_ne = {}
    for var in ['total_infected_+20', 'total_infected_-20']:
        data_groups = [sensitivity_ne[sensitivity_ne['scenario'] == s][var] for s in
scenarios if not sensitivity_ne[sensitivity_ne['scenario'] == s].empty]
        if len(data_groups) == len(scenarios):
            h_stat, p_val = stats.kruskal(*data_groups)
            kw_sens_ne[var] = {'H': round(h_stat, 3), 'p': f"{p_val:.2e}"}

    print("\nSensitivity Kruskal-Wallis Results (New England Only):")
    print(kw_sens_ne)

    sensitivity_melted_ne = pd.melt(sensitivity_ne, id_vars=['scenario'],
value_vars=['total_infected', 'total_infected_+20', 'total_infected_-20'],
                                 var_name='variation', value_name='infected_count')

    plt.figure(figsize=(10, 6))
    sns.barplot(x='scenario', y='infected_count', hue='variation',
data=sensitivity_melted_ne)
    plt.title('Sensitivity Analysis: Impact of ±20% Transmission Rate (New England Only)')
```

```
plt.xlabel('Scenario')
plt.ylabel('Infected Premises')
plt.grid(True, axis='y', linestyle='--', alpha=0.7)
max_sens_y_ne = sensitivity_melted_ne['infected_count'].max()
plt.yticks(np.arange(0, max_sens_y_ne + 5, 5))
plt.tight_layout()
plt.savefig('sensitivity_NE1D_analysisIQR_ne.png')
plt.close()
```

2. FIGURE S1:

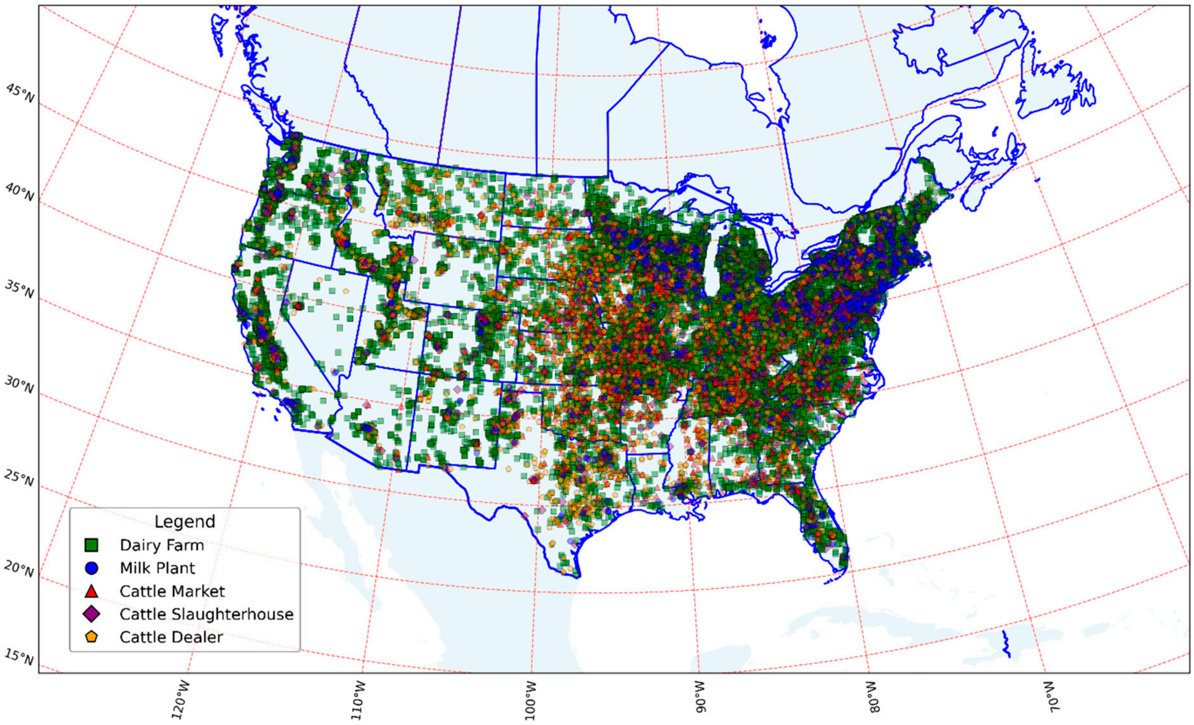

Figure S1: Showing the USA Contiguous Albers Equal Area Conic Map of Premises Locations
